# Hypophosphatemia in Patients With Multiple Myeloma

**DOI:** 10.7759/cureus.40487

**Published:** 2023-06-15

**Authors:** Ivan Cancarevic, Usman Ilyas, Mahmoud Nassar

**Affiliations:** 1 Internal Medicine, Icahn School of Medicine at Mount Sinai, Queens Hospital Center, New York, USA; 2 Internal Medicine, Icahn School of Medicine at Mount Sinai, NYC Health + Hospitals/Queens, New York, USA

**Keywords:** electrolyte, electrolyte disturbances, multiple myeloma, phosphorus, hypophosphatemia

## Abstract

Hypophosphatemia is among the most common electrolyte abnormalities, especially among patients with underlying malignancies, and is frequently associated with adverse prognoses. Phosphorus levels are regulated through a number of mechanisms, including parathyroid hormone (PTH), fibroblast growth factor-23 (FGF-23), vitamin D, and other electrolyte levels themselves. Clinically, the findings are nonspecific, and the diagnosis is frequently delayed. This article is a narrative literature review. The PubMed database was searched for relevant articles pertaining to hypophosphatemia causes and consequences in patients suffering from multiple myeloma. We found a variety of causes of hypophosphatemia in patients with multiple myeloma. Tumor-induced osteopenia, although more common among patients with small squamous cell carcinomas, can occur with multiple myeloma as well. Additionally, both light chains themselves and medications can trigger Fanconi syndrome, which leads to phosphorus wasting by the kidney. Bisphosphonates, in addition to being a possible cause of Fanconi syndrome, lead to a decrease in calcium levels, which then stimulates parathyroid hormone (PTH) release, predisposing the patient to significant hypophosphatemia. Additionally, many of the more modern medications used to manage multiple myeloma have been associated with hypophosphatemia. A better understanding of those mechanisms may give clinicians a clearer idea of which patients may need more frequent screening as well as what the potential triggers in the individual patient may be.

## Introduction and background

Hypophosphatemia, generally defined as a serum phosphate level of less than 2.5 mg/dL, is a common clinical entity affecting a number of organ systems [1]. The majority of body phosphorus is stored in the bone; however, phosphate is also an essential component of adenosine triphosphate (ATP), as well as several other molecules essential for metabolism and energy generation [1,2]. It is mostly absorbed from the gastrointestinal (GI) tract, while further regulation of phosphorus balance is mostly achieved by the kidneys [2]. While less is known about phosphate regulation than about calcium regulation, several regulatory mechanisms have been described, including vitamin D, fibroblast growth factor-23 (FGF-23), parathyroid hormone (PTH), and serum calcium and phosphorus levels themselves [2,3]. Therefore, any disease process that affects any of the aforementioned regulatory hormones or molecules could feasibly lead to difficult-to-control phosphorus disorders. Moreover, any factor leading to increased losses, whether renal or extrarenal or decreased intake or absorption of phosphorus, can lead to overall phosphorus depletion [3].

Clinical manifestations of hypophosphatemia are largely nonspecific and in milder cases include weakness, either due to rhabdomyolysis or neuropathy, while in severe cases may include encephalopathy and impaired cardiac function, including life-threatening arrhythmias. However, those generally do not occur unless phosphorus levels drop below 0.3 mg/dL [2,4]. Neuropathy associated with hypophosphatemia can be severe and, with decreasing phosphorus levels, ultimately lead to paralysis, which tends to be reversible once phosphate has been repleted [5]. A recent systematic review found a significant association between hypophosphatemia and cardiomyopathy development, with left ventricular function readily improving after the correction of severe, but not mild, hypophosphatemia [6]. Mild deficiencies are typically corrected by increasing dietary phosphate or providing oral supplementation, while more severe hypophosphatemia tends to require intravenous repletion to avoid life-threatening consequences [7]. Multiple studies have looked at the prognostic implications of hypophosphatemia. A large meta-analysis among intensive care unit (ICU) patients found that hypophosphatemia was associated with increased lengths of both ICU and hospital stays, but not with mortality [8]. Even when looking at individual conditions, such as alcoholic pancreatitis or COVID-19, hypophosphatemia also appears to be associated with poorer overall outcomes [9,10].

Multiple myeloma is a common hematologic malignancy characterized by a range of cytogenetic abnormalities that eventually lead to the proliferation of monoclonal plasma cells and the secretion of monoclonal antibodies [11]. Part of the pathology of multiple myeloma includes increased osteoclastic bone resorption and decreased bone formation with myeloma cells being found in the areas of bone resorption [12,13]. More specifically, factors such as receptor activator of nuclear factor kappa-B ligand (RANKL) stimulate osteoclasts, while Dickkopf-related protein 1 (DKK1) inhibits the activity of osteoblasts [13]. The combination of those processes leads to characteristic radiographic findings and an increased risk of fractures. In addition, hypercalcemia is one of the hallmark findings of multiple myeloma. In those patients, it is usually treated with bisphosphonates, and hypophosphatemia is a known side effect of them [14,15]. Hypercalcemia, however, can cause the suppression of PTH release, possibly predisposing the patient to hyperphosphatemia. On the other hand, there are studies reporting the coexistence of hyperparathyroidism and multiple myeloma, including some that suggest a possible link between the two [16-18]. Calcium can also chelate phosphate.

Overall, multiple myeloma has the potential to affect phosphorus levels through a variety of pathways and mechanisms. Hypophosphatemia, due to its nonspecific early presentation and frequent lack of inclusion of phosphorus among routine chemistries, may often go unnoticed until it becomes severely symptomatic, at which point it is also very dangerous. Considering the potential risks associated with hypophosphatemia, a deeper understanding of the mechanisms that lead to its development in multiple myeloma patients could provide insight into which patients are at especially high risk and in whom routine phosphate monitoring may be indicated, adding an important layer to the comprehensive care of those patients.

In this article, we are going to review the currently available literature using the PubMed database for the association between multiple myeloma and hypophosphatemia. Combinations of the following keywords were used for the search: “multiple myeloma,” “hypophosphatemia,” “tumor-induced osteomalacia,” “bisphosphonates,” “Fanconi syndrome,” “carfilzomib,” “ixazomib,” “elotuzumab,” and “daratumumab.”

## Review

Overview of hypophosphatemia in hematology and oncology

Hypophosphatemia is common in cancer patients and can result from cancer’s effects on the kidney or bone, a paraneoplastic syndrome, or as a side effect of therapy (e.g., some medications can lead to Fanconi syndrome) [19]. Nearly half of cancer patients may be at risk for hypophosphatemia, with nearly half of these cases being severe hypophosphatemia with phosphorus levels of less than 2.0 mg/dL [20]. A large study of over 25,000 patients found that hypophosphatemia was the second most common electrolyte derangement in cancer patients after hypocalcemia and affected over 25% of them [21].

Extensive abdominal surgeries are frequently performed for both primary and metastatic malignancies. Hypophosphatemia has been associated with surgical resection of the liver and pancreas [22,23]. According to Wong et al. [22], almost half of all the patients who underwent pancreatectomies developed hypophosphatemia. Significant hypophosphatemia frequently develops after hepatectomies and laparotomies without resection, according to Zheng et al. [23]. Proposed mechanisms have involved phosphorus usage by regenerating organs and urinary loss of phosphorus [22]. Paraneoplastic syndromes can also cause clinically significant decreases in phosphate levels. One of the best-known paraneoplastic syndromes is the secretion of parathyroid hormone-related peptide (PTHrP) in patients with squamous cell carcinoma of the lung [24]. PTHrP leads to decreased reabsorption of phosphate in renal tubules leading to phosphate wasting and hypophosphatemia [25].

Fibroblast growth factor-23

Fibroblast growth factor (FGF) undeniably plays a major role in phosphate metabolism. Osteocytes secrete FGF and act on the distal nephron and parathyroid gland [26]. It primarily acts to increase phosphate excretion by stimulating the internalization of the sodium-phosphate co-transporters in the kidney, preventing reabsorption, although other effects, including opposing ones, have been proposed [26]. Additionally, it downregulates 1-alpha-hydroxylase, halting the conversion of 25-hydroxycholecalciferol into 1,25-dihydroxycholecalciferol, which, consequently, decreases phosphate absorption from the gastrointestinal tract [26]. Finally, it directly binds receptors found in the parathyroid gland, decreasing the secretion of parathyroid hormone (PTH) [26]. The regulation of FGF-23 is complex and involves a number of factors, many of which are still poorly understood [26-28]. A number of diseases with impaired FGF-23 regulation have been described, such as osteoglophonic dysplasia, where fibroblast growth factor receptor 1 (FGFR1)-activating mutation leads to increased FGF-23 production or familial tumoral calcinosis, where a mutation limiting the cleavage of FGF-23 leads to ectopic calcifications [28]. In advanced renal diseases, FGF-23 levels rise significantly, often as high as 200 times the normal limit [28].

Moreover, some authors believe that FGF-23 can be used clinically as an early marker of renal bone disease; however, this has not become standard practice at the time of this writing [29]. However, recently, more is known about the role of FGF-23 in other conditions. Its role in cardiorenal interactions in heart failure appears limited, and a recent study of 199 patients failed to demonstrate any significant correlation between FGF-23 and cardiorenal parameters [30]. When it comes to diabetes, the association is complex. Several studies point toward independent associations between FGF-23 and insulin resistance, obesity, and inflammation, regardless of renal function [31,32]. Moreover, multiple studies suggest that higher FGF-23 levels are associated with adverse outcomes in patients with diabetes with or without concomitant chronic kidney disease [33,34].

Tumor-induced osteomalacia in multiple myeloma patients

**Figure 1:**
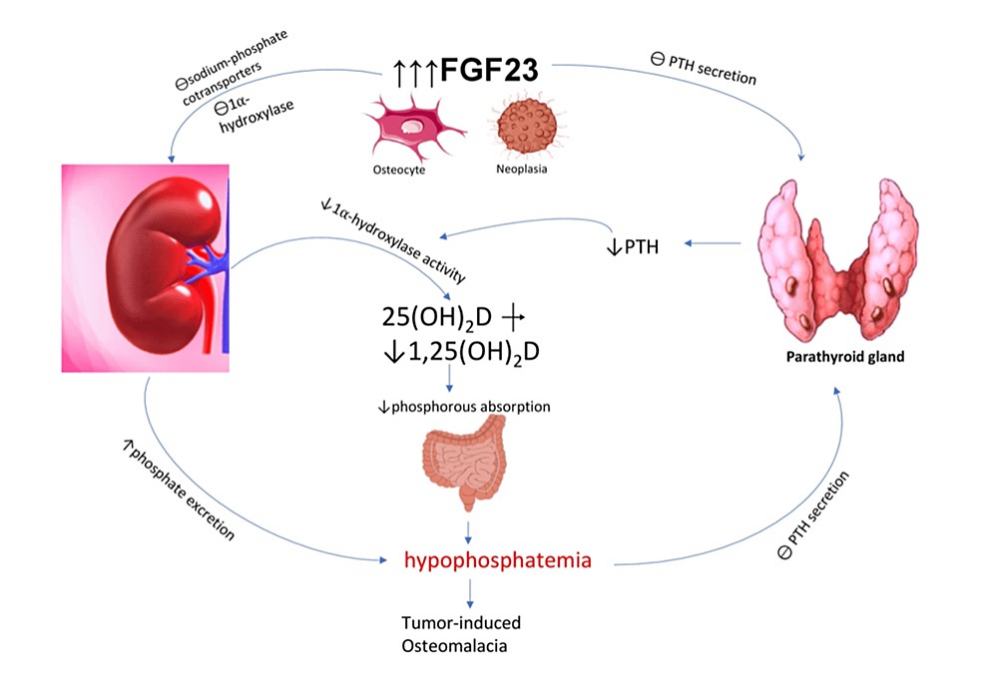
Tumor-induced osteomalacia FGF-23: fibroblast growth factor-23, PTH: parathyroid hormone

Tumor-induced osteomalacia (TIO) is an uncommon paraneoplastic syndrome characterized by overproduction of FGF-23 by the tumor as well as inappropriately normal or low levels of vitamin D due to impaired hydroxylation leading to phosphate wasting by the kidney and eventual hypophosphatemia (Figure 1) [35-40]. Most cases of TIO are caused by small, slow-growing mesenchymal tumors that can be difficult to visualize, locate, and, consequently, excise [36]. Due to the nonspecific symptoms of hypophosphatemia, the slow-growing features of the tumors, and their relatively small size, hypophosphatemia may persist for years, allowing for overt osteomalacia to develop [37]. The diagnosis frequently requires many modalities besides the standard computerized tomography (CT) and/or magnetic resonance imaging (MRI), such as positron emission tomography (PET), octreotide scintigraphy, and venous sampling with FGF-23 measurements [38-40]. Although the exact prevalence of TIO is difficult to determine, a recent study from Denmark [41] found the prevalence of TIO to be no higher than 0.43 in 100,000 adults. Crotti et al. [42] reported their experience in a single tertiary care center involving 17 patients, 10 females and seven males. The phosphorus levels were 1.3 ± 0.4 mg/dL, while FGF-23 levels were 358.9 ± 677 pg/mL (reference range: 25-45 pg/mL) [42]. Reabsorption of phosphorus in renal tubules was decreased in all 17 patients; interestingly, only two patients had elevated levels of urinary phosphorus [42].

Reports of TIO in patients with various hematologic malignancies, including multiple myeloma, are scarce but nevertheless present [43-46]. Narvaez et al. [44] in 2005 postulated that the pathophysiology of hypophosphatemic osteomalacia in most myeloma patients differed from that in other malignancies and was mostly related to light chain nephropathy causing tubular damage and phosphorus wasting in the absence of Fanconi syndrome. However, Stewart et al. [46] found FGF-23 to be elevated in several cases of multiple myeloma and monoclonal gammopathy of undetermined significance (MGUS). Furthermore, it appears that multiple myeloma cells themselves express receptors for FGF-23 [47]. Notably, despite having elevated levels of FGF-23, in Stewart et al. [46], those patients were not hypophosphatemic, either because the FGF-23 secreted by plasma cells varied in some way from the FGF-23 secreted by other tumors or because of other effects myeloma has on phosphorus metabolism. Those findings may align with Narvaez et al.’s proposed mechanism; however, notably, FGF-23 levels were not reported in the Narvaez et al.’s study. More recently, Lin and Ganda [48] reported a more typical case of tumor-induced osteomalacia with elevated FGF-23 and significant hypophosphatemia. However, a question remains on whether, in the absence of those compensatory mechanisms, multiple myeloma and MGUS could lead to significant TIO. It is certain, however, that the relationship between plasma cell disorders and FGF-23 is complex and insufficiently understood. Improved understanding of those mechanisms could potentially significantly decrease morbidity associated with bone disease of multiple myeloma, especially considering that Terzi Demirsoy et al. [49] found that patients with elevated FGF-23 levels had decreased overall survival, indicating that FGF-23 either does have a role in the pathogenesis of myeloma or that it could simply be a marker of more advanced disease.

Fanconi syndrome in patients with multiple myeloma

**Figure 2:**
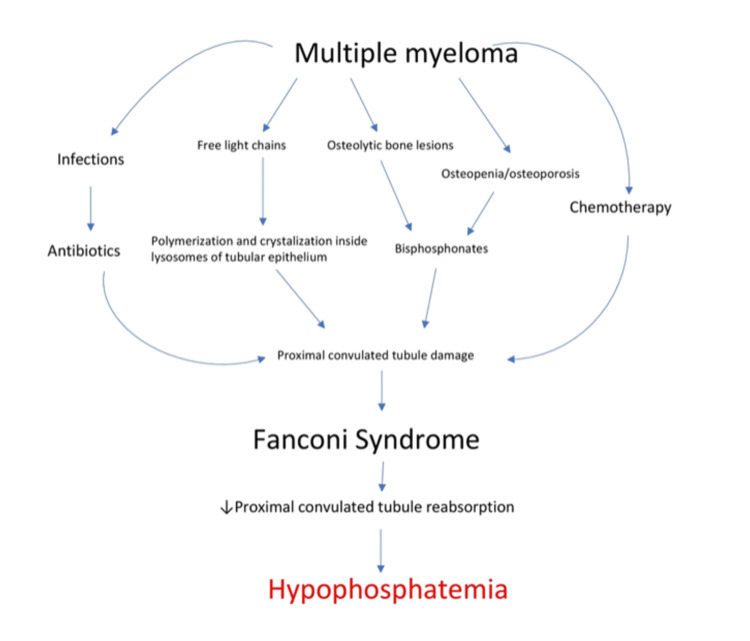
Multiple myeloma-induced Fanconi syndrome

Fanconi syndrome, a condition involving proximal tubules of the nephron, is characterized by renal wasting of several substrates, including phosphorus [49]. It can be triggered by various medications, including antibiotics, chemotherapies, and primary conditions, such as hematologic malignancies (Figure 2) [19,50]. Smaller studies have shown that Fanconi syndrome-induced hypophosphatemia can be significant enough to lead to osteomalacia [51]. A study of four patients found that in patients with Fanconi syndrome, FGF-23 levels are not necessarily associated with vitamin D and phosphate levels [52]. Light chains are low-molecular-weight proteins that the glomerulus can filter. In monoclonal gammopathies, the amount of protein the renal tubules are exposed to can be significant enough to cause tubular damage (Figure 2). Light chains undergo polymerization inside the lysosomes of tubular epithelium forming crystals, leading to lysosomal dysfunction and impaired reabsorption [53,54]. There is evidence that when Fanconi syndrome does develop in these patients, it occurs early in the course of the disease, during the MGUS or smoldering multiple myeloma (SMM) phase [55]. Currently, there is a lack of definitive evidence that chemotherapy is effective in treating Fanconi syndrome in this setting; however, Wu et al. reported that bortezomib may be effective [55,56].

Additionally, Fanconi syndrome is frequently associated with the use of chemotherapeutics (Figure 2). One of the drugs most commonly associated with Fanconi syndrome is ifosfamide [57,58]. Although it is commonly used in children, it has also occasionally been used as part of multidrug regimens in advanced myeloma [57-60]. Another drug frequently associated with Fanconi syndrome is cisplatin [61]. Although more frequently thought of as a drug used for solid malignancies, cisplatin has also been used for multiple myeloma [62]. Myeloma patients are also more prone to infectious complications and are more frequently prescribed antimicrobial therapies, especially broad-spectrum antibiotics, compared to the general population. Aminoglycosides are known to be nephrotoxic, and one of the common manifestations of that nephrotoxicity is the development of Fanconi syndrome [63]. There are also reports of other antibiotics causing Fanconi syndrome [64,65]. It can also be triggered by various other medications [66,67].

Bisphosphonates-induced hypophosphatemia in multiple myeloma

Bisphosphonates, such as alendronate or zoledronate, are the most commonly prescribed treatments for osteoporosis or other diseases characterized by increased bone turnover [68,69]. Mainly, they act by inhibiting osteoclast function, which prevents bone resorption [69,70]. Their effect on fracture healing appears to be minimal [69,70]. The efficacy of bisphosphonates at decreasing morbidity from any form of bone tumor, including solid tumor metastases and multiple myeloma, is well documented [71]. Moreover, since several chemotherapeutic treatments cause considerable side effects (such as ovarian failure), which could potentially increase the risk of osteoporosis, bisphosphonates can also be used in settings other than direct involvement of the bone [71]. Two newer studies even suggest that they may have a role in preventing bone metastasis in patients with breast cancer, although using them for the said purpose is still not a standard practice, and more evidence will be needed [72,73]. Additionally, they are used to treat hypercalcemia, including hypercalcemia of malignancy [74,75]. There are several indications for bisphosphonate use in multiple myeloma, including osteolytic bone lesions and hypercalcemia (hallmark features of multiple myeloma), and studies suggest overall decreased morbidity in patients treated with bisphosphonates [76].

**Figure 3:**
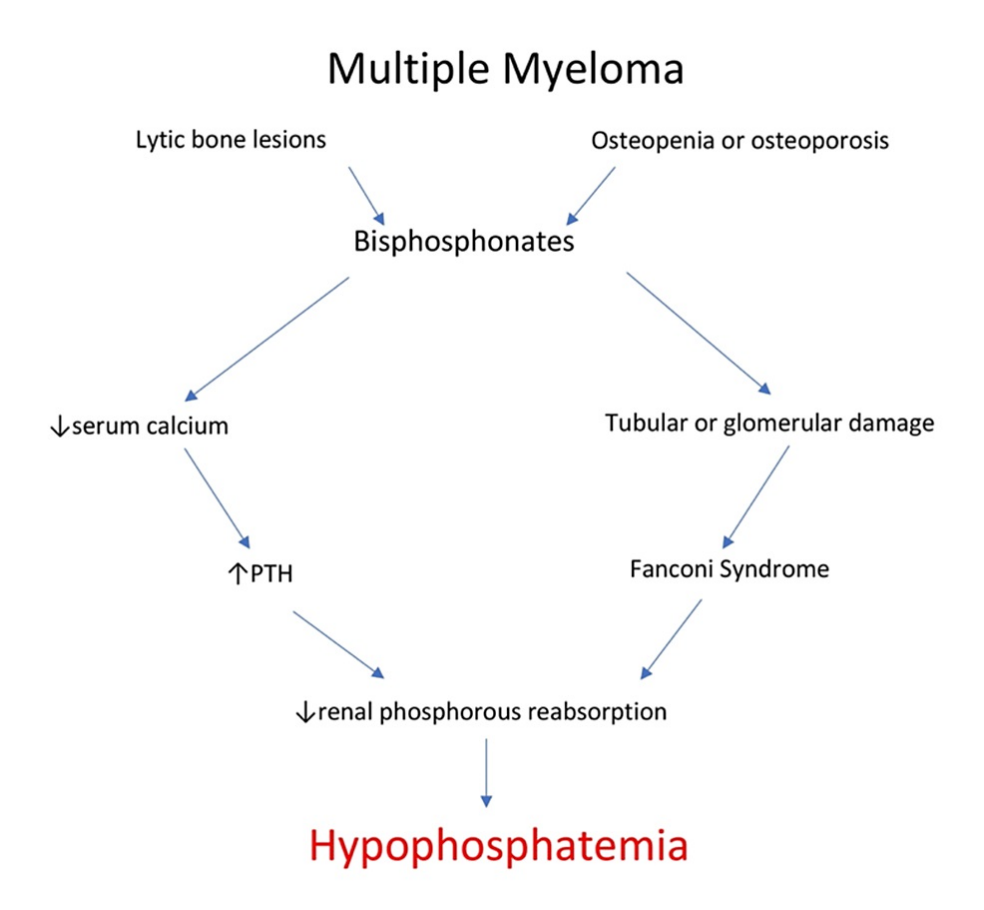
Bisphosphonate-induced hypophosphatemia PTH: parathyroid hormone

While osteonecrosis of the jaw is probably the most characteristic and well-known side effect of bisphosphonates, nephrotoxicity and hypophosphatemia are also important and frequently limiting [77]. Kidney damage occurs in the form of tubular and/or glomerular damage [77]. In some cases, proximal tubular damage can be severe enough for Fanconi syndrome to develop [78]. Fanconi syndrome, as described previously, can lead to profound hypophosphatemia. Additionally, a calcium level drop can trigger a cascade that eventually results in hypophosphatemia (Figure 3). Decreased calcium levels are a trigger to increase the secretion of parathyroid hormone (PTH), which subsequently induces hypophosphatemia [15]. Hypophosphatemia induced by bisphosphonates can be severe [79-81].

Other medication-induced hypophosphatemia in multiple myeloma

Monoclonal antibodies are becoming an increasingly important aspect of cancer management, and multiple of them have been used to treat multiple myeloma. A phase II study recently evaluated the safety and efficacy of adding elotuzumab in patients with refractory or relapsed myeloma and found it to be effective and overall well tolerated [82]. Notably, over 40% of patients developed some degree of hypophosphatemia, while 15% developed significant hypophosphatemia (classified as grade 3 or higher according to the Common Terminology Criteria for Adverse Events) [82]. This confirmed earlier findings from a single-center study where all patients treated with elotuzumab experienced decreasing phosphorus levels with decreases as low as 44% below the baseline [83]. While a question of which medication in a combination regimen (especially dexamethasone with elotuzumab) is the direct cause of hypophosphatemia, the same study concluded that it was likely associated with elotuzumab in particular because the only patients who developed hypophosphatemia were those treated with elotuzumab and no cases were observed among those treated with dexamethasone alone [83]. Similarly, a phase II study that evaluated daratumumab in patients with relapsed and/or refractory multiple myeloma found grade 3-4 hypophosphatemia to be a possible side effect [84].

Proteasome inhibitors are also commonly used in advanced myeloma. Carfilzomib, a parenteral medication, causes hypophosphatemia in as many as 25% of patients who are started on it [85]. Ixazomib, frequently combined with dexamethasone, is an oral proteasome inhibitor that has also been associated with hypophosphatemia in multiple studies, although significant hypophosphatemia appears to occur less frequently (in around 4% of patients) compared to carfilzomib or monoclonal antibodies [86,87]. Even bortezomib, previously mentioned as a possible treatment for Fanconi syndrome, has been associated with hypophosphatemia [88].

It should, however, be noted that in all the cases referenced previously, the proteasome inhibitors and monoclonal antibodies were combined with other treatments, including immunomodulators and corticosteroids, raising the possibility of confounding effects or synergy. Corticosteroids such as dexamethasone, in particular, may contribute to hypophosphatemia as they increase urinary phosphorus excretion [89]. A recent study by Bosman et al. [90] found that 16% of patients suffering from Cushing syndrome had hypophosphatemia before treatment. They also found a negative correlation between cortisol and phosphate levels, and once the disease was in remission, phosphate levels increased, confirming findings from some previous case reports [90]. However, the molecular understanding of corticosteroids’ role in phosphorus metabolism remains inadequate. As previously noted, in one study, no multiple myeloma patients treated with dexamethasone only developed hypophosphatemia [83].

Spurious hypophosphatemia in multiple myeloma

In multiple myeloma patients, spurious hypophosphatemia due to paraprotein interference with laboratory assays should be considered as a differential diagnosis, particularly in patients with asymptomatic severe hypophosphatemia, persistent hypophosphatemia despite repeated repletion, or impaired GFR with hypophosphatemia, as phosphate tends to accumulate due to decreased renal excretion [91,92]. Mechanisms of paraprotein interference with the phosphate assay include direct bindings to inorganic phosphate, protein precipitation in distilled water, or interference with the formation and stabilization of phosphomolybdate or chromogenic substances in many automatic auto-analyzers [91]. Most phosphate assays use ammonium molybdate to form a phosphomolybdate complex, the concentration of which is either measured directly or after adding a reducing agent to obtain a stable color that is measured calorimetrically [91]. In both these assays, the sample is highly diluted with the addition of assay reagents; therefore, any interfering substance is decreased in concentration [92]. In most cases, simple serial dilution is sufficient to avoid interference, except for patients with high concentrations of immunoglobulins [93,94].

In contrast, in the manual method, the addition of trichloroacetic acid precipitates and removes plasma proteins before the phosphate assay, thereby obliterating interference [92]. Therefore, analysis of the phosphate concentration using a laboratory method that employs sample deproteinization or ultrafiltration should be used to exclude the possibility of spurious hypophosphatemia [94,95]. It is imperative to be aware of this phenomenon to avoid unnecessary extensive diagnostic procedures and risky treatment, as intravenous phosphate repletion can result in hypocalcemia or devastating precipitation of calcium phosphate complexes in soft tissues [94].

## Conclusions

Multiple myeloma, through various effects on bone, predisposes patients to significant phosphate derangements, including hypophosphatemia, predisposing patients to significant cardiac and neurological morbidity. Currently, there is a lot of evidence that hypophosphatemia worsens clinical outcomes. Fibroblast growth factor-23 is a known regulator of phosphorus levels, and impaired regulation of it has been associated with a condition known as tumor-induced osteomalacia. While it is more common with some mesenchymal tumors, it has also been found in patients with multiple myeloma. It is difficult to manage, and early recognition is important. Furthermore, both the myeloma itself and its therapies predispose patients to develop renal tubular damage, including Fanconi syndrome, characterized by phosphorus wasting. Other drugs, such as antibiotics, which are frequently required in the setting of myeloma-induced immunosuppression, may further increase the risk of Fanconi syndrome. Bisphosphonates are commonly used in multiple myeloma and can also cause hypophosphatemia. Hypophosphatemia has also been observed as a side effect in a number of early clinical trials of novel monoclonal antibodies and proteasome inhibitors, although more data will need to be collected before clearer conclusions are reached regarding those medications.
